# Candida chorioretinitis in renal transplant recipient with candidemia related to contaminated organ preservation fluid: A role for dilated fundus examination in its management

**DOI:** 10.1016/j.idcr.2023.e01793

**Published:** 2023-05-08

**Authors:** Raphaël Schils, Radhika Rampat, Jean-Marie Rakic, François-Xavier Crahay

**Affiliations:** a Department of Internal Medicine and Infectious Diseases, University of Liege, Liege, Belgium; b Department of Ophtalmology, Queen Victoria Hospital NHS Foundation, East Grinstead, United Kingdom; c Department of Ophtalmology, University of Liege, Liege, Belgium; d Department of Ophtalmology, Hopital de la Citadelle, Liege, Belgium

**Keywords:** Fundus, Candidemia, Chorioretinitis, Preservation fluid, Transplantation

## Abstract

Infection by *Candida* spp is a potentially life-threatening condition among both immunocompromised and immunocompetent patients. *Candida* chorioretinitis can occur as a complication of candidemia and may develop into endophthalmitis if not detected and treated early, which can lead to irreversible visual loss. Here, we report on a 52-year-old diabetic woman who developed candidemia complicated by bilateral chorioretinitis following kidney transplantation. Antifungal therapy was immediately started but fundoscopic examination highlighted multiple bilateral chorioretinal lesions. Given new onset of vomiting and increased number of retinal lesions on repeat fundus examination a few weeks later, the patient underwent a positron emission tomography (PET) which revealed a mycotic arterial pseudoaneurysm at the renal graft anastomosis. It led ineluctably to transplantectomy, aneurysm flattening and vascular reconstruction a few days later. Blood cultures remained negative and fundus examination progressively showed a regression of chorioretinal lesions until their complete disappearance a few months later. Our case emphasizes the importance of a non-invasive examination which allowed to accelerate and optimize in a consequential way the management of the patient leading to her recovery after a long antifungal treatment.

## Introduction

Candidemia is a rare but potentially dramatic complication especially in immunocompromised patients such as kidney transplant recipients.*.* Its incidence as a nosocomial infection has progressively increased in the last 20 years [1]. Conversely, the rate of ocular candidiasis has decreased to an incidence of less than 2% [2]. Although not frequent, ocular candidiasis can have disastrous visual consequences such as blindness. It can manifest as a spectrum of chorioretinitis, vitritis or even endophthalmitis. The evolution is slowly progressive and may remain asymptomatic (or undetectable) at the beginning as some critically ill patients are unable to communicate or even being aware of their visual status. Risk factors include intravenous catheters, malignancy, neutropenia, broad-spectrum antibiotics, glucocorticoid therapy, abdominal surgery and intravenous hyperalimentation [3], [4].

We report on a 52-year-old woman with recent kidney transplant who developed candidemia complicated by bilateral chorioretinitis. The likely cause of candidemia is the contaminated preservation fluid (PF) by *Candida albicans*. Infection was perpetuated by the mycotic aneurysm leading to refractory ocular lesions that persisted despite prompt fluconazole therapy. After transplant removal, the chorioretinal lesions progressively decreased in size and number. Here we demonstrate the importance of dilated ocular fundus examinations in monitoring of a patient diagnosed with candidemia, especially after an organ transplant.

## Case presentation

A 52-year-old woman with type II diabetes mellitus and hypertension developed end-stage renal disease requiring kidney transplantation. In addition, she had a background of hypertensive and proliferative diabetic retinopathy with diabetic maculopathy.

Unfortunately, *Candida albicans* was detected in the donor kidney PF in small quantities and twice in the transplanted kidney biopsy. In the context of immunosuppressive treatment, lack of graft function recovery and histological signs of graft rejection, urgent intravenous fluconazole was started (800 mg the first day then 400 mg daily). The glomerular filtration rate was estimated under 15 mL/min/1.73 m² at this clinical point.

The patient developed asymptomatic candidemia 12 days after transplantation. Antifungal susceptibility tests confirmed sensitivity to fluconazole with a minimum inhibitory concentration (MIC) of 0.25 mg/L. A concomitant abdominal wall abscess caused by *Candida albicans* was also discovered by performing CT scan and therefore immediately drained.

**Fig. 1:**
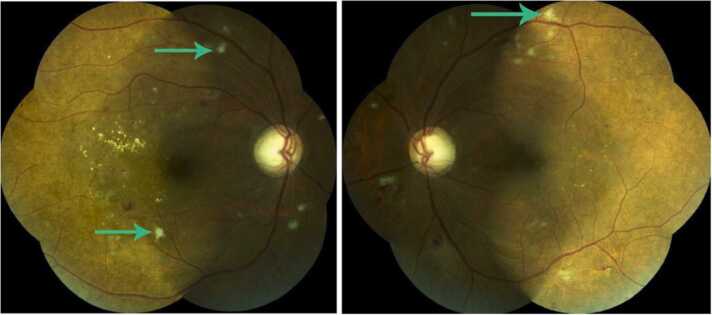
Colour fundus photographs of the right and the left eye: the arrows show creamy-white, cotton-like retinal lesions that are compatible with *Candida* chorioretinis.

Despite a 48 h duration of candidemia, a principled ophthalmic examination was performed to rule out ocular involvement after two weeks of treatment by intravenous fluconazole. Best-corrected visual acuity was 6/6 in both eyes with the Snellen visual acuity chart. The patient had no visual complaints and anterior segment assessment with slit lamp biomicroscopy was normal without any signs of inflammation. Dilated fundus examination demonstrated bilateral creamy-white, well-circumscribed small cotton-like intra-retinal lesions near the main retinal vessels (Fig. 1) suggesting a fungal chorioretinitis.

This was in addition to diabetic maculopathy and retinopathy (hard exudates, hemorrhages). The diagnosis was also supported by fluoangiography and optic coherence tomography findings. Magnetic resonance imaging (MRI) of the brain ruled out the presence of abscesses. The patient also underwent angio-MRI of the renal arteries which highlighted infiltration of the graft sinus fat without any signs of pyelonephritis or arterial aneurysm. Oral fluconazole 400 mg was then given to allow the patient to leave the hospital while the function of the graft had recovered satisfactorily and her general condition was good.

**Fig. 2:**
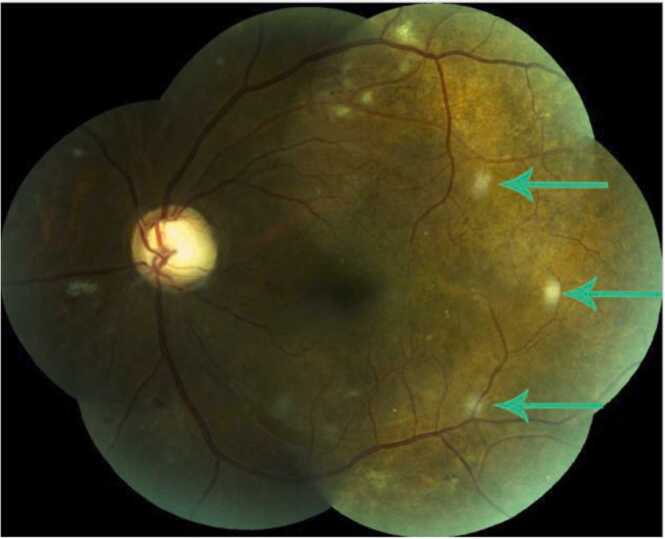
Left eye fundus photograph demonstrates new lesions (arrows).

Six weeks after antifungal therapy initiation, an increased number of lesions were found on repeat fundus examination (Fig. 2) motivating a new hospitalization. The patient remained afebrile but her general condition finally deteriorated with the presence of vomiting without abdominal pain. Serum fluconazole concentration reached 10 mg/L (N: 0.5–6 mg/L). No endocarditis was detected on cardiac trans-esophageal echography but PET highlighted a mycotic arterial pseudoaneurysm at the renal graft anastomosis. The patient therefore underwent transplantectomy, flattening of the aneurysm and vascular reconstruction of the artery with a femoral venous graft. Cultures yielded *Candida albicans* with the same sensitivity to fluconazole which was maintained**.**

**Fig. 3:**
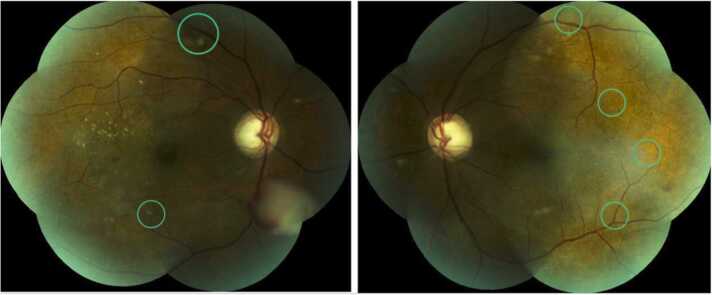
Right and left eye fundus photographs after transplantectomy and continued fluconazole treatment. The lesions are flatter, less active with a cicatricial appearance (especially on left eye).

Eight days after surgery, retinal lesions decreased for the first time. At the two-month follow up, the number of lesions had decreased further and the remaining lesions were visibly flatter, less active, more akin to scar like lesions (Fig. 3).

PET performed 3 months after transplantectomy showed complete regression of vascular hypermetabolism and a persistent abdominal wall abscess which motivated the continuation of antifungal therapy for 6 months until complete radiological resolution of the abscess. Eight months later, no new retinal lesion were detected.

## Discussion

Firstly, this case highlights the need for vigilance in the conditions in which organs are harvested, conserved, prepared and screened. Fungal contamination of PF generally occurs during graft harvesting. Among the fungi, *Candida albicans* is the most common pathogen associated with graft-site arteritis [5]. The majority of cases of donor-derived candidiasis involved kidney transplant recipients in whom contaminated PF is a frequently suspected source. These donor-derived fungal infections can potentially trigger a mycotic pseudoaneurysm whose most feared complication is anastomotic rupture [6]. The incidence of fungal PF contamination in kidney transplant varies between 0.86% and 14.4% [7], [8], [9], [10]. However, incidence of mycotic pseudoaneurysm remains rare (< 1%) but has led American Society of Transplantation to recommend pre-emptive antifungal therapy when PF cultures yield *Candida* [11], [12], [13].

Secondly, although some studies based on epidemiologic data suggested that the management of candidemia patients without fundus examination is safe [14], our case demonstrated the grave importance of a systematic dilated fundus examination for both the diagnosis of chorioretinitis and follow up [15]. Eye findings compatible with candidiasis in patients with candidemia can confirm the diagnosis without vitreous culture [4]. It is important to note that the only examination that led us to suspect the mycotic arterial pseudo-aneurysm was the worsening of the fundus appearance as the blood cultures quickly became negative. If the fundus had not been performed, a low-noise progression of this aneurysm would have been possible with a non-negligible risk of rupture and hemorrhagic shock. Just after transplantectomy, fundus lesions decreased proving the treatment’s efficacy.

In conclusion, *candida* chorioretinitis can be completely asymptomatic. Our case underlines the importance of systematic and repeated fundus examinations in patients with candidemia. In case of ocular candidiasis, repeated fundus examinations are useful to follow up and to confirm treatment’s efficacy. We believe that the benefit of early identification of retinal lesions during candidemia and thus prevention of visual loss outweighs the cost of performing a fundus examination.

## Consent for publication

Written informed consent was obtained from the patient for publication of this case report and accompanying images. A copy of the written consent is available for review by the Editor-in-Chief of this journal on request.

## Ethical approval

Not needed.

## Geographical information

Liège (4000), Belgium.

## Funding

There is no funding to report for this manuscript.

## Declaration of Competing Interest

The authors report no declarations of interest.
